# Liver fibrosis is closely related to metabolic factors in metabolic associated fatty liver disease with hepatitis B virus infection

**DOI:** 10.1038/s41598-023-28351-3

**Published:** 2023-01-25

**Authors:** Haifeng Lv, Yanming Jiang, Geli Zhu, Shiyi Liu, Dian Wang, Jie Wang, Ke Zhao, Jing Liu

**Affiliations:** 1 Department of Intensive Care Unit, The First Affiliated Hospital, Zhejiang University School of Medicine, Hangzhou, 310000 Zhejiang China; 2 Department of Hepatology, The Affiliated Hospital of Hangzhou Normal University, No. 126 Wenzhou Road, Gongshu District, Hangzhou, 310015 Zhejiang China; 3 Zhejiang Chinese Medical University, Hangzhou, 310035 Zhejiang China; 4 School of Clinical Medicine, Hangzhou Normal University, Hangzhou, 310015 Zhejiang China; 5 School of Basic Medical Sciences, Hangzhou Normal University, Hangzhou, 311121 Zhejiang China

**Keywords:** Infectious diseases, Metabolic disorders, Hepatology

## Abstract

This case–control study aimed to identify the clinical characteristics and explore the risk factors for liver fibrosis in metabolic associated fatty liver disease (MAFLD) patients with hepatitis B virus (HBV) infection. The patients were grouped into MAFLD + HBV and MAFLD (without HBV infection). Propensity score matching (PSM) was used to match baseline features between the groups. We included 401 patients with biopsy-proven MAFLD, 179 of whom had HBV infection. A total of 83 pairs were successfully matched via PSM, and steatosis scores and ballooning in the MAFLD + HBV group were lower than those in the MAFLD group, while the inflammation scores and liver fibrosis stages were higher. After adjusted for confounding factors, HBV infection was associated with a higher risk of significant liver fibrosis in patients with MAFLD [odds ratio (OR): 3.140, *P* = 0.003]. Overall, 43.58% (78/179) of patients in the MAFLD + HBV group had significant liver fibrosis. Further multivariate regression analysis, hypertension (OR: 2.640; *P* = 0.031)*,* type 2 diabetes (OR: 4.939; *P* = 0.035), and elevated glutamyl-transferase levels (OR: 3.980; *P* = 0.001) were risk factors for liver fibrosis in the MAFLD + HBV group. This suggests metabolic rather than viral factors are more closely associated with liver fibrosis in MAFLD patients with HBV infection.

## Introduction

Over the past 20 years, under the influence of overnutrition and sedentary lifestyle in modern society, non-alcoholic fatty liver disease (NAFLD) has increased rapidly, posing a major health and economic burden to all societies^1,2^. In 2020, the International Fatty Liver Expert Group announced that NAFLD was renamed as metabolic dysfunction-associated fatty liver disease (MAFLD). Both metabolic disorders and hepatic steatosis are necessary for the diagnosis of MAFLD^3^, but unlike NAFLD, does not require the exclusion of other defined etiologies of chronic liver disease, such as viral infection or excessive alcohol intake^4,5^.

Hepatitis B virus (HBV) infections are common in Asia. The prevalence of HBV infection is approximately 5%-6% in the general Chinese population^6^. Therefore, a large population is potentially at risk of developing MAFLD with concurrent HBV infection, forming an important subtype of MAFLD. The coexistence of metabolic dysfunction and viral infection is a striking feature of MAFLD complicated by HBV infection, which may act synergistically to significantly increase the risk of cirrhosis, hepatocellular carcinoma (HCC), and liver-related deaths^7–10^.

The progression of liver fibrosis has been generally accepted as a reliable factor for predicting the overall or liver-related death rate among MAFLD cases^11–14^. However, the clinical characteristics and risk factors for liver fibrosis in MAFLD patients infected with HBV remain unclear, and whether metabolic or viral factors were more closely related to liver fibrosis is also currently unknown. To address these issues, we compared the metabolic, etiological, and histological features of MAFLD patients with and without HBV infection in a large biopsy-proven cohort, and then explored the risk factors of liver fibrosis in MAFLD patients infected with HBV.

## Methods

### Study subjects and design

**Figure 1:**
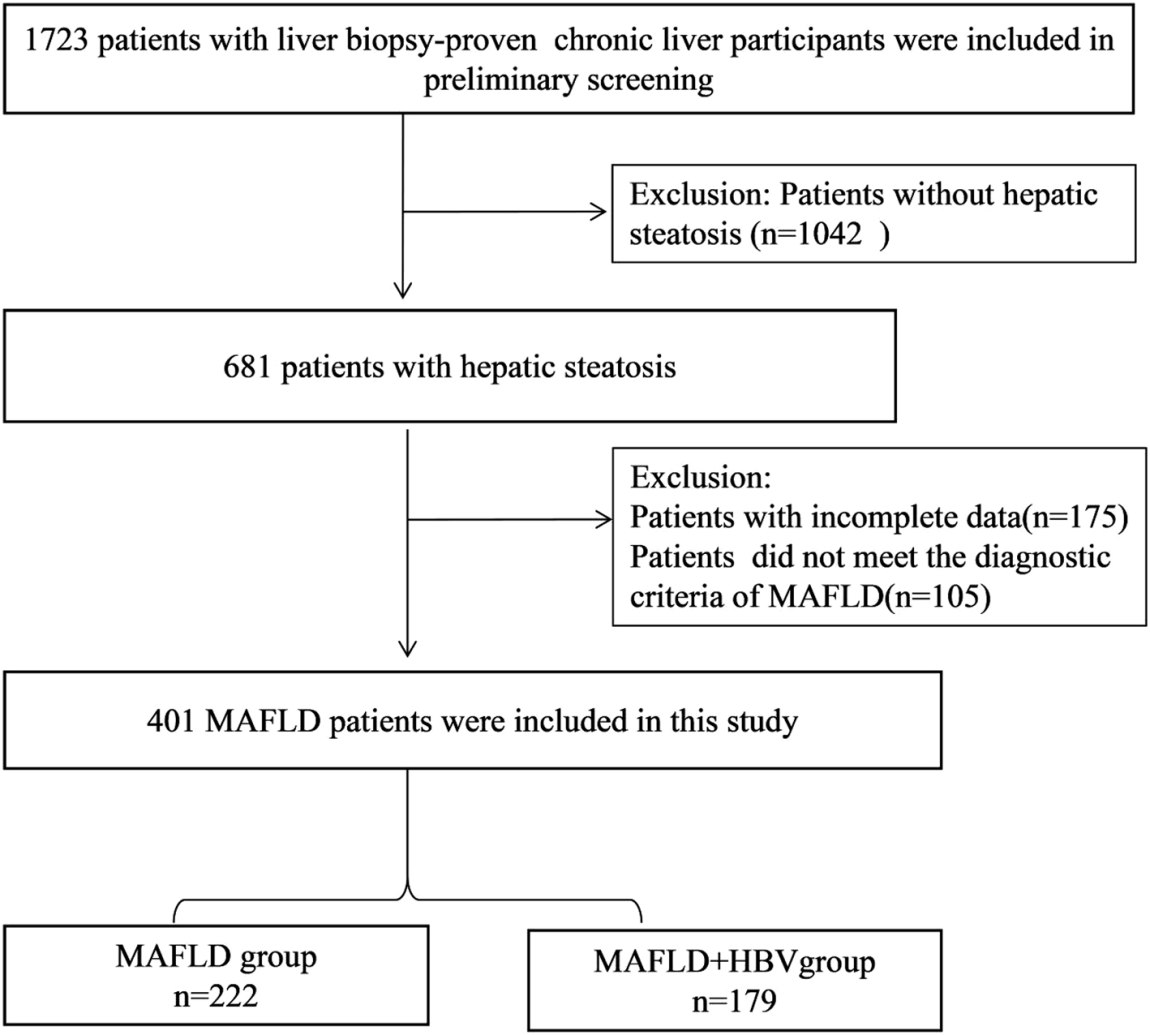
Design of the cross-sectional study. MAFLD, metabolic dysfunction-associated fatty liver disease.

This cross-sectional study included all patients with liver stiffness measurements greater than 7 kPa as determined by Fibroscan prior to liver biopsy between 2011 and 2021 at the Affiliated Hospital of Hangzhou Normal University (Hangzhou, China). Individuals with the following conditions were excluded (Fig. 1): (1) evidence of chronic liver diseases such as chronic viral hepatitis C, alcoholic liver disease, autoimmune liver disease; (2) history of malignancy or missing data concerning weight, heigh, or etiological markers, including hepatitis B surface antigen (HBsAg), hepatitis B e-Antigen (HBeAg), and hepatitis B virus deoxyribonucleic acid (HBV DNA); (3) use of hypoglycemic or antilipidemic drugs exerting potential effects on liver fibrosis; (4) patients with hepatitis B receiving antiviral therapy.

This study conformed to the ethical guidelines of the Declaration of Helsinki and was approved by the Ethics Committee of the Affiliated Hospital of Hangzhou Normal University (Approval Number/ID: 2020 (02)-KS-022). As this was an observational retrospective study, the requirement for informed consent was waived by the Ethics Committee.

### Clinical examination, biochemical analyses, and biopsy assessment

Diastolic blood pressure (DBP), systolic blood pressure (SBP), height, and body weight were measured by professional physicians in accordance with standard protocols. Body mass index (BMI) was calculated as weight (kg) divided by height (m) squared (kg/m^2^). Blood samples were obtained after 8 h of fasting, and routine blood and biochemical tests were conducted to assess the following: fasting plasma glucose (FPG), triglycerides (TG), total cholesterol (TC), high/low-density lipoprotein cholesterol (HDL-c/LDL-c), alanine aminotransferase (ALT), gamma-glutamyl-transferase (GGT), aspartate aminotransferase (AST), albumin (ALB), glycated hemoglobin (HbA1c), and serum uric acid (SUA). The biochemical tests were performed using an automated biochemical analyzer in accordance with the manufacturer’s instructions (Model 7180; Hitachi, Tokyo, Japan). Serological markers of HBV infection, including HBsAg, HBcAb, HBeAg, and hepatitis B e-Antibody (anti-HBe), were obtained using commercially available enzyme immunoassays. Serum HBV DNA levels were quantified using a commercially available real-time polymerase chain reaction assay in accordance with the manufacturer’s instructions, with a linear dynamic detection range of 3 × 10^1^–10 × 10^9^ IU/ml.

All liver biopsies were reassessed by three experienced histopathologists blinded to participant details. The steatosis score (positive if > 5%, according to the Brunt classification), stage of fibrosis (based on a meta-analysis of histological data for viral hepatitis score), ballooning, and degree of inflammation were evaluated ^15–17^. Fibrosis stage ≥ 2, degree of inflammation ≥ 2, and steatosis score ≥ 2 was defined as significant liver fibrosis, active inflammation, and severe steatosis, respectively.

### Diagnosis

The diagnosis of MAFLD was based on evidence of hepatic steatosis, which was determined according to the results of liver biopsy in patients with BMI values ≥ 25, those with type 2 diabetes mellitus (T2DM), or those with at least two metabolic risk abnormalities despite BMI values lower than 25 ^5^. Abnormalities indicative of metabolic risk included the following: (1) TG ≥ 1.70 mmol/L or use of certain medications; (2) HDL-c < 1.0 for men and 1.3 mmol/L for women or use of certain medications; (3) BP ≥ 130/85 mmHg or use of certain medications; (4) prediabetes FPG 5.6–6.9 mmol/L, HbA1c 5.7–6.4%, or 2-h post-load glucose level 7.8–11.0 mmol/L; and (5) waist circumference more than 90 cm and 80 cm in men and women, respectively. Data for high-sensitivity C-reactive protein (SCRP) and homeostasis model assessment index insulin resistance (HOMA-IR) were absent. Patients with MAFLD were divided into two groups based on the results of the HBsAg test: MAFLD + HBV group (HBsAg positive) and MAFLD group (HBsAg negative). In terms of disease course, HBV infection was diagnosed by histopathologists based on the location of inflammation and immunohistochemical results.

### Statistical analysis

Continuous variables were analyzed via Student’s t-test or the Mann–Whitney U-test when compared between two groups. The chi-square test was used to compare categorical variables. Propensity score matching (PSM) was used to balance age, sex, and metabolic factors between the two groups at a ratio of 1:1 and using a caliper value of 0.2. Univariate and multivariate logistic regression analyses were performed to identify factors contributing to liver fibrosis. Adjusted odds ratios (OR) and relevant 95% confidence intervals (CIs) were estimated using a parametric proportional hazards model. SPSS version 26.0 (IBM, Armonk, NY, USA) was used for statistical analyses, with *P* < 0.05 indicating statistical significance.

### Disclosure of ethical statement

This study conformed to the ethical guidelines of the Declaration of Helsinki and was approved by the Ethics Committee of the Affiliated Hospital of Hangzhou Normal University (Approval Number/ID: 2020 (02)-KS-022).

### Consent to participate/consent to publish

As this was an observational retrospective study, the requirement for informed consent was waived by the Ethics Committee.

## Results

### Establishment of the study

As shown in Fig. 1, a total of 681 patients with biopsy-proven steatosis were included in the data screening. A total of 175 individuals with steatosis were excluded since they did not meet the diagnostic criteria for MAFLD, while 105 individuals were further excluded due to incomplete data. Among the 401 patients diagnosed with MAFLD, 256 patients were overweight/obese, 64 patients had T2DM, and 81 patients with normal or lean weight had metabolic disorders. The average age of the included patients was 43.14 ± 11.31 years; 44.64% (179/401) of patients had MAFLD with HBV infection (MAFLD + HBV group), and 55.36% (222/401) of patients had MAFLD without HBV infection (MAFLD group).

The proportion of male patients was higher in the MAFLD + HBV group than in the MAFLD group (88.82% vs. 72.52%, P < 0.001). No significant differences in age, SBP, DBP, BMI, FPG, HDL-c, SUA, or ALB levels were observed between the MAFLD and MAFLD + HBV groups (*P* > 0.05). However, the MAFLD + HBV group exhibited lower levels of liver enzymes, LDL-c, TG, and TC, as well as lower rates of obesity, hypertension, T2DM, low HDL-c, hypertriglyceridemia, hypercholesterolemia, and hyperuricemia than the MAFLD group (*P* < 0.05) (Table 1).

**Table 1:** Comparison of clinical characteristics between metabolic dysfunction-associated fatty liver disease with and without hepatitis B virus infection before and after propensity score matching.

Variables	Before propensity score matching			After propensity score matching (1:1)		
	MAFLD	MAFLD + HBV	*P*-value	MAFLD	MAFLD + HBV	*P*-value
n (male)	222 (161)	179 (159)	< 0.001^‡^	83 (70)	83 (70)	1.000^‡^
Age (year)	43.50 ± 12.34	42.68 ± 9.91	0.47	44.35 ± 13.30	42.70 ± 10.31	0.373
BMI (kg/m^2^)	27.73 ± 4.85	26.05 ± 2.89	0.336	26.81 ± 3.59	27.11 ± 2.89	0.554
SBP (mmHg)	132.65 ± 15.15	131.19 ± 14.47	0.333	131.24 ± 17.10	132.51 ± 13.30	0.61
DBP (mmHg)	82.00 ± 11.45	81.44 ± 10.34	0.618	80.64 ± 11.19	81.43 ± 10.43	0.636
SUA (μmol/L)	391.68 ± 122.20	371.90 ± 86.95	0.077	381.60 ± 114.01	370.77 ± 92.42	0.515
FPG (mmol/L)	6.14 ± 1.63	5.82 ± 1.80	0.067	5.71 ± 0.95	6.04 ± 2.20	0.206
HbA1c	6.18 ± 1.13	6.25 ± 1.81	0.788	5.98 ± 1.15	6.57 ± 1.90	0.148
TG (mmol/L)	2.02 (1.38–2.84)	1.08 (1.55–2.18)	< 0.001	1.73 (1.19–2.34)	1.71 (1.20–2.59)	0.851
TC (mmol/L)	5.05 ± 1.79	4.65 ± 0.89	< 0.001	4.65 ± 1.02	4.75 ± 0.99	0.543
LDL-c (mmol/L)	3.17 ± 1.46	2.87 ± 0.72	0.012	2.87 ± 0.76	2.94 ± 0.80	0.54
HDL-c (mmol/L)	1.13 ± 0.27	1.10 ± 0.26	0.23	1.09 ± 0.24	1.09 ± 0.28	0.857
ALT (U/L)	41.00 (29.00–63.00)	58.00 (36.00–86.00)	0.002	56.00 (38.00–103.00)	64.00 (37.00–98.00)	0.801
AST (U/L)	66.00 (41.00–114.00)	35.00 (26.00–49.00)	0.041	38.00 (26.00–53.00)	37.00 (26.00–59.00)	0.732
GGT (U/L)	62.00 (36.00–118.00)	40.00 (28.00–60.00)	< 0.001	50.00 (33.00–84.00)	47.00 (32.00–66.00)	0.385
ALB (g/L)	45.05 ± 4.87	45.02 ± 6.50	0.954	45.18 ± 4.29	44.81 ± 8.89	0.731
Obesity (n, %)	147 (66.22)	86 (48.04)	< 0.001^‡^	58 (69.88)	67 (80.72)	0.105^‡^
Hypertension (n, %)	123 (55.41)	58 (32.40)	< 0.001^‡^	32 (38.55)	36 (43.37)	0.528^‡^
T2DM (n, %)	46 (20.72)	18 (10.11)	0.004^‡^	8 (9.64)	11 (13.25)	0.465^‡^
Low HDL-c (n, %)	80 (36.04)	36 (20.11)	< 0.001^‡^	33 (39.76)	41 (49.40)	0.212^‡^
Hypertriglyceridemia (n, %)	141 (63.51)	76 (42.46)	< 0.001^‡^	42 (50.60)	43 (51.80)	0.887^‡^
Hypercholesterolemia (n, %)	62 (27.93)	19 (10.61)	< 0.001^‡^	14 (16.87)	11 (13.25)	0.515^‡^
Hyperuricemia (n, %)	83 (37.39)	42 (23.46)	0.003^‡^	26 (31.33)	20 (24.10)	0.370^‡^

Data are expressed as mean ± standard deviation or median (interquartile range).

*MAFLD* metabolic dysfunction-associated fatty liver disease, *BMI* body mass index, *SBP* systolic blood pressure, *DBP* diastolic blood pressure, *SUA* serum uric acid, *FPG* fasting plasma glucose, *TG* triglycerides, *TC* total cholesterol, *LDL-c* low-density lipoprotein cholesterol, *HDL-c* high-density lipoprotein cholesterol, *ALT* alanine aminotransferase, *AST* aspartate aminotransferase, *GGT* gamma-glutamyl-transferase, *T2DM* type 2 diabetes mellitus.

After PSM, 83 pairs were finally matched, and there were no statistically significant differences in sex, age, BMI, DBP, SBP, FPG, LDL-c, TC, liver enzyme levels, or rates of hypertension, T2DM, low HDL-c, hypertriglyceridemia, hypercholesterolemia, or hyperuricemia between the two groups (*P* > 0.05) (Table 1).

### Comparison of histological features in MAFLD patients with and without HBV infection

**Figure 2:**
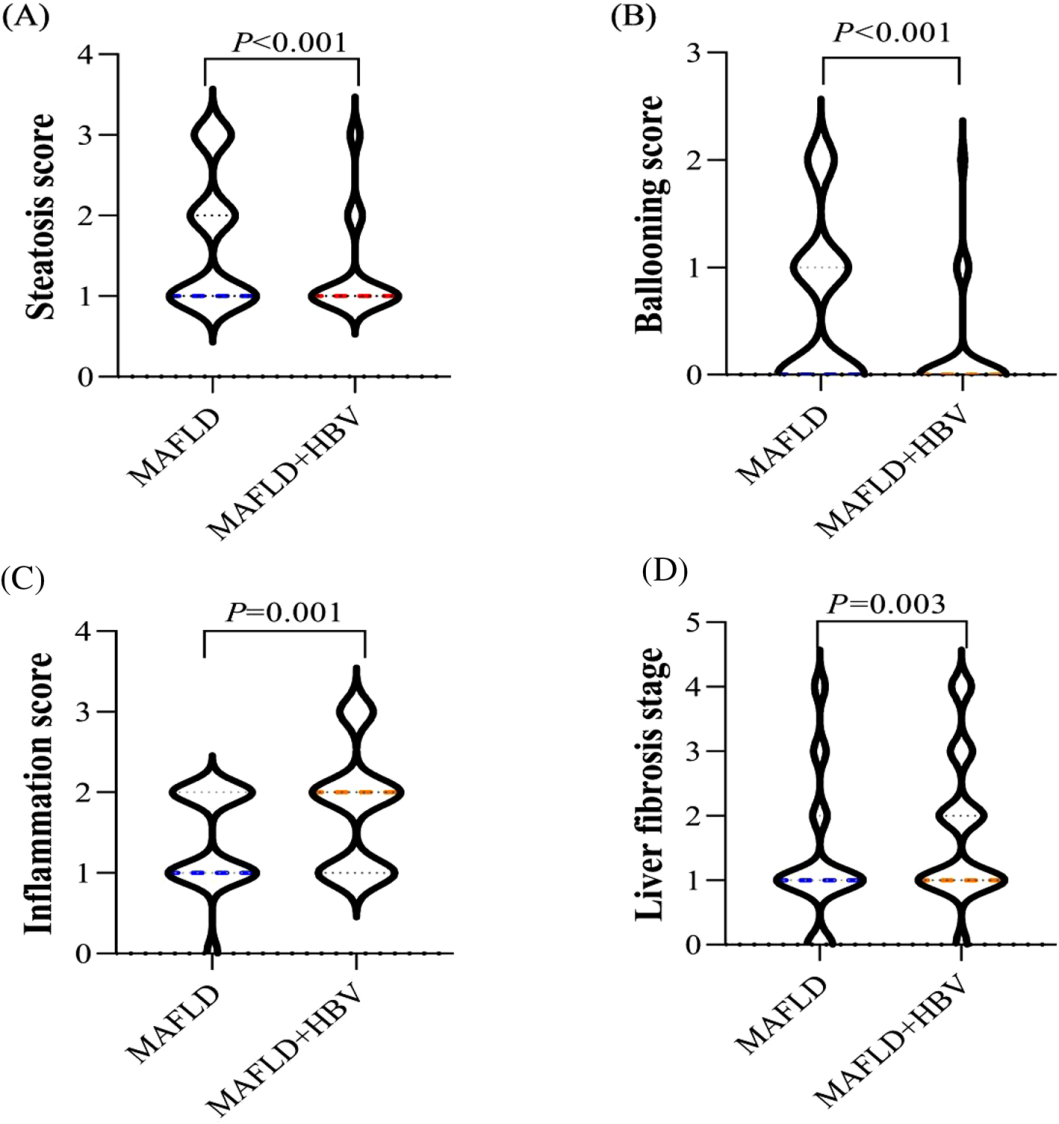
Comparison of histopathological characteristics between metabolic dysfunction-associated fatty liver disease with and without hepatitis B virus (HBV) infection. (**A**) Comparison of hepatic steatosis score segregated by HBV infection; (**B**) comparison of ballooning score segregated by HBV infection; (**C**) comparison of inflammation score segregated by HBV infection; (**D**) comparison of fibrosis stage segregated by HBV infection.

After PSM, sex, age, BMI, liver enzymes, and metabolic factors were comparable between patients with and without HBV infection. Inflammation scores and liver fibrosis stages were higher in the MAFLD + HBV group than in the MAFLD group, while steatosis and ballooning scores were lower (*P* < 0.05) (Fig. 2). In the multivariate analysis, model 1 was adjusted for age and sex, while model 2 was adjusted for model 1 plus metabolic parameters, including BMI, T2DM, low HDL-c, hypertriglyceridemia, hypercholesterolemia, high LDL-c, hypertension, and hyperuricemia. The results indicated that HBV infection was associated with lower hepatic steatosis scores (OR: 0.251, 95% CI: 0.117–0.542, *P* < 0.001) and ballooning scores (OR: 0.119, 95% CI: 0.049–0.294, *P* < 0.001) yet higher stages of liver fibrosis (OR: 3.140, 95% CI: 1.479–6.663, *P* = 0.003) in patients with MAFLD (Table 2). However, no significant differences were observed in inflammation between patients with and without HBV infection after adjusting for confounders.

**Table 2:** Odds ratio of hepatitis B virus infection for liver fibrosis in metabolic dysfunction-associated fatty liver disease population.

	Crude		Model 1		Model 2	
	OR (95% CI)	*P*-value	OR (95% CI)	*P*-value	OR (95% CI)	*P*-value
Severe steatosis						
MAFLD	Ref		Ref		Ref	
MAFLD + HBV	0.298 (0.152–0.586)	< 0.001	0.293 (0.148–0.578)	< 0.001	0.251 (0.117–0.542)	< 0.001
Ballooning						
MAFLD	Ref		Ref		Ref	
MAFLD + HBV	0.200 (0.096–0.415)	< 0.001	0.202 (0.097–0.423)	< 0.001	0.119 (0.049–0.294)	< 0.001
Active inflammation						
MAFLD	Ref		Ref		Ref	
MAFLD + HBV	1.981 (1.067–3.678)	0.03	1.972 (1.060–3.666)	0.032	1.803 (0.925–3.514)	0.083
Liver fibrosis						
MAFLD	Ref		Ref		Ref	
MAFLD + HBV	2.617 (1.358–5.044)	0.004	2.817 (1.438–5.518)	0.003	3.140 (1.479–6.663)	0.003

Model 1 adjusted for age and sex; Model 2 was adjusted for model 1 plus BMI, hypertension, type 2 diabetes mellitus, hypertriglyceridemia, hypercholesterolemia, hyperuricemia, high LDL-c, and low HDL-c.

*MAFLD* metabolic dysfunction-associated fatty liver disease, *HBV* hepatitis B virus, *OR* odds ratio, *CI* confidence interval.

### Comparison of clinical and histological features of patients with and without liver fibrosis in MAFLD infected with HBV

Of the 179 patients in MAFLD + HBV group, 18.44% (33/179) of patients had HBeAg-positive chronic infection, 27.37% (49/179) of patients had HBeAg-positive chronic hepatitis, 22.91% (41/179) of patients had HBeAg-negative chronic infection, and 31.28% (56/179) had HBeAg-negative chronic hepatitis. Further, 75.98% (136/179) of patients were HBV-DNA-positive and 43.58% (78/179) of patients in the MAFLD + HBV group had significant liver fibrosis (S ≥ 2). There were no statistically significant differences in sex, age, SBP, DBP, BMI, ALT, AST, LDL-c, HDL-c, TG, or TC between patients with and without liver fibrosis. In addition, rates of HBeAg-positive, HBV-DNA-positive, hypertension, low HDL-c, hypertriglyceridemia, hypercholesterolemia, and hyperuricemia did not significantly differ between patients with and without liver fibrosis (*P* > 0.05). Among patients in MAFLD + HBV group, those with liver fibrosis had higher levels of GGT and FPG, and a higher percentage of T2DM and obese/overweight status than those without (*P* < 0.05) (Table 3). For histological comparison, the proportion of patients with active inflammation (inflammation score ≥ 2) was significantly higher among those with liver fibrosis than those without liver fibrosis (85.90% vs. 37.62%, *P* < 0.001), whereas the proportions of severe steatosis and ballooning degeneration had no significant difference between the two groups (*P* > 0.05).

**Table 3:** Comparison of clinical characteristics among MAFLD patients with hepatitis B virus infection according to the presence of significant liver fibrosis.

Variables	With fibrosis	Without fibrosis	*P*-value
	N = 78	N = 101	
Male (n, %)	70 (89.74)	89 (88.12)	0.732^‡^
Age (year)	41.68 ± 9.70	43.79 ± 10.10	0.126
BMI (kg/m^2^)	25.53 ± 2.58	26.73 ± 3.14	0.016
SBP (mmHg)	130.46 ± 15.20	132.12 ± 13.53	0.458
DBP (mmHg)	80.67 ± 11.09	82.43 ± 9.27	0.271
SUA (μmol/L)	366.25 ± 74.41	379.26 ± 101.05	0.338
FPG (mmol/L)	5.52 ± 1.00	6.21 ± 2.44	0.013
HbA1c	6.02 ± 1.70	6.48 ± 1.95	0.462
TG (mmol/L)	1.45 (1.08–2.25)	1.57 (1.08–2.18)	0.591
TC (mmol/L)	4.62 ± 0.81	4.69 ± 0.99	0.611
LDL-c (mmol/L)	2.86 ± 0.65	2.87 ± 0.81	0.475
HDL-c (mmol/L)	1.08 ± 0.25	1.11 ± 0.27	0.847
ALT (U/L)	70.00 (37.00–89.00)	56.00 (35.00–81.00)	0.128
AST (U/L)	37.00 (27.00–59.00)	31.00 (25.00–46.00)	0.06
GGT (U/L)	53.00 (32.00–77.00)	34.00 (24.00–52.00)	< 0.001
ALB (g/L)	45.18 ± 2.61	44.79 ± 9.49	0.693
Etiology			
Disease course			
Chronic infection			
HBeAg positive (n, %)	5 (33.33)	28 (47.46)	0.236^‡^
HBeAg negative (n, %)	10 (66.67)	31 (52.54)	
Chronic hepatitis			
HBeAg positive (n, %)	29 (46.03)	20 (47.62)	0.873^‡^
HBeAg negative (n, %)	34 (53.97)	22 (52.38)	
HBV DNA			
Positive (n, %)	56 (71.79)	80 (79.21)	0.250^‡^
Negative (n, %)	22 (28.21)	21 (20.79)	
Metabolic dysfunction			
Obesity/overweight (n, %)	54 (69.23)	71 (70.30)	0.878^‡^
Hypertension (n, %)	29 (37.18)	29 (28.71)	0.230^‡^
T2DM (n, %)	14 (17.94)	4 (3.96)	0.002^‡^
High LDL-c (n, %)	15 (19.23)	21 (20.79)	0.796^‡^
Low HDL-c (n, %)	33 (42.31)	45 (44.55)	0.764^‡^
Hypertriglyceridemia (n, %)	31 (39.74)	45 (44.55)	0.518^‡^
Hypercholesterolemia (n, %)	9 (11.54)	10 (9.90)	0.724^‡^
Hyperuricemia (n, %)	23 (29.49)	19 (18.81)	0.095^‡^
Histology			
Hepatic steatosis			
< 2 (n, %)	62 (79.49)	89 (88.12)	0.115^‡^
≥ 2 (n, %)	16 (20.51)	12 (11.88)	
Inflammation			
< 2 (n, %)	11 (14.10)	63 (62.38)	< 0.001^‡^
≥ 2 (n, %)	67 (85.90)	38 (37.62)	
Ballooning			
< 1 (n, %)	69 (88.46)	91 (90.10)	0.724^‡^
≥ 1 (n, %)	9 (11.54)	10 (9.90)	

Data are expressed as mean ± standard deviation or median (interquartile range).

*MAFLD* metabolic dysfunction-associated fatty liver disease, *BMI* body mass index, *SBP* systolic blood pressure, *DBP* diastolic blood pressure, *SUA* serum uric acid, *FPG* fasting plasma glucose, *TG* triglycerides, *TC* total cholesterol, *LDL-c* low-density lipoprotein cholesterol, *HDL-c* high-density lipoprotein cholesterol, *ALT* alanine aminotransferase, *AST* aspartate aminotransferase, *GGT* gamma-glutamyl-transferase, *HBeAg* hepatitis B e-antigen, *HBV DNA* hepatitis B virus deoxyribonucleic acid, *T2DM* type 2 diabetes mellitus.

### Risk factors for liver fibrosis in MAFLD patients with HBV infection

Univariate and multivariate logistic regression analyses were performed to further explore risk factors for liver fibrosis in MAFLD patients with HBV infection. Univariate analysis confirmed that T2DM (OR: 5.540, 95% CI: 1.72–17.63; *P* = 0.004) and elevated GGT levels (OR: 2.991, 95% CI: 1.612–5.550; *P* = 0.001) were risk factors for liver fibrosis (Table 4). Multivariate regression analysis, with sex, age, metabolic factors, viral factors, and liver enzymes taken into consideration, revealed that hypertension (OR: 2.640, 95% CI: 1.091–6.368; *P* = 0.031), T2DM (OR: 4.939, 95% CI: 1.121–21.796; *P* = 0.035), and elevated GGT levels (OR: 3.980, 95% CI: 1.735–9.132; *P* = 0.001) were independent risk factors for liver fibrosis in MAFLD patients with HBV infection.

**Table 4:** Risk factors of liver fibrosis in metabolic dysfunction-associated fatty liver disease patients with hepatitis B virus infection.

Variables	Univariate analysis		Multivariate analysis	
	OR (95% CI)	*P*-value	OR (95% CI)	*P*-value
Old age				
No	Ref			
Yes	2.194 (0.689–6.993)	0.184	–	–
Male				
No	Ref			
Yes	1.180 (0.457–3.044)	0.732	–	–
BMI > 23 kg/m^2^				
No	Ref			
Yes	1.141 (0.307–4.244)	0.844	–	–
Hypertension				
No	Ref			
Yes	1.479 (0.786–2.782)	0.225	2.640 (1.091–6.368)	0.031
T2DM				
No	Ref			
Yes	5.540 (1.72–17.63)	0.004	4.939 (1.121–21.796)	0.035
Hypertriglyceridemia				
No	Ref			
Yes	0.821 (0.451–1.495)	0.591	–	–
Hypercholesterolemia				
No	Ref			
Yes	1.187 (0.458–3.079)	0.725	–	–
Hyperuricemia				
No	Ref			
Yes	1.840 (0.909–3.723)	0.9	–	–
High LDL-c				
No	Ref			
Yes	0.913 (0.503–1.657)	0.764	–	–
Low HDL-c				
No	Ref			
Yes	0.907 (0.433–1.902)	0.796	–	–
HBeAg positive				
No	Ref			
Yes	0.853 (0.461–1.545)	0.6	–	–
HBV DNA positive				
No	Ref			
Yes	0.700 (0.323–1.516)	0.366	–	–
Elevated ALT				
No	Ref			
Yes	1.271 (0.662–2.441)	0.471	–	–
Elevated AST				
No	Ref			
Yes	1.457 (0.794–2.673)	0.224	–	–
Elevated GGT				
No	Ref			
Yes	2.991 (1.612–5.550)	0.001	3.980 (1.735–9.132)	0.001

*BMI* body mass index, *T2DM* type 2 diabetes mellitus, *LDL-c* low-density lipoprotein cholesterol, *HDL-c* high-density lipoprotein cholesterol, *ALT* alanine aminotransferase, *AST* aspartate aminotransferase, *HBeAg* hepatitis B e-Antigen, *HBV DNA* hepatitis B virus deoxyribonucleic acid, *GGT* gamma-glutamyl-transferase, *OR* odds ratio, *CI* confidence interval.

## Discussion

MAFLD with HBV infection is a distinct subtype of MAFLD in which metabolic and viral factors co-exist. The current results indicate that the presence of HBV is associated with lower steatosis scores and ballooning grades but a higher liver fibrosis stage in patients with MAFLD. Further risk factor analysis for liver fibrosis revealed that T2DM, hypertension, and elevated GGT levels were independent risk factors for liver fibrosis in MAFLD patients with HBV infection.

MAFLD patients with HBV infection exhibit unique histopathological characteristics (Fig. 2). Our findings are in accordance with a recent study showing that, although HBV infection is associated with a lower degree of steatosis and ballooning, it independently increases the risk of liver fibrosis in patients with MAFLD^18,19^. In our study, HBV infection resulted in a threefold increase in the risk of significant liver fibrosis in patients with MAFLD (Table 2). Therefore, for MAFLD patients with HBV infection, early screening and intervention for risk factors of liver fibrosis are required, as liver fibrosis has been identified as an accelerator for cirrhosis and hepatocellular carcinoma in chronic liver disease^20^.

Previous studies have reported that metabolic disorders such as T2DM, hypertension, dyslipidemia, and obesity are closely related to NAFLD liver fibrosis^21–26^. HBeAg negativity is associated with more advanced liver fibrosis in patients with chronic hepatitis B^27^. ALT and AST have also been identified as excellent predictors of significant liver fibrosis in patients with CHB^28^. The presence of both viral and metabolic factors may accelerate disease progression in MAFLD patients with HBV infection. Our study indicates that T2DM, hypertension, and elevated GGT levels are independent risk factors for significant liver fibrosis in MAFLD patients with HBV infection even after adjusting for confounding factors (Table 4). While HBeAg positivity and HBV-DNA positivity were not associated with liver fibrosis in MAFLD patients with HBV. It suggested that metabolic factors is more associated with liver fibrosis compared with viral factors in MAFLD patients with HBV infection.

This study highlights that T2DM, hypertension, and elevated GGT levels are closely associated with liver fibrosis in MAFLD patients with HBV infection. Liver fibrosis is the result of an excessive production of extracellular matrix (ECM) that is not adequately maintained, resulting in net accumulation. In the liver, hepatic stellate cells (HSCs) constitute the main source of ECM-producing fibroblasts in models of toxic and biliary liver disease and NAFLD^29,30^. Insulin resistance (IR) in T2DM is recognized as an integral component of NAFLD pathogenesis that worsens with disease progression^29,31,32^ and the activation of HSC by IR is largely divided into distinct direct and indirect pathways. The renin–angiotensin–aldosterone system is well recognized for its essential role in the physiological regulation of blood volume, blood pressure, and sodium homeostasis^33,34^. Increasing evidence demonstrates that this system is overactive at different stages of liver fibrosis^33,35^, which may explain the association between hypertension and liver fibrosis. As a surface enzyme, GGT can cleave extracellular glutathione (GSH), maintain the balance of GSH in vivo, and play a key role in alleviating the effects of oxidative stress^36^. Previous studies confirmed that elevated GGT was associated with SCRP, low adiponectin, the presence of chronic kidney disease, and hepatic steatosis. It was reported that GGT elevation was associated with hepatic steatosis, and fibrosis in patients with NAFLD^37,38^. In addition to the above factors, the influence of genetic factors such as PNPLA3, TM6SF2, and MBOAT7 on liver fibrosis in MAFLD patients with HBV infection should also be further studied^39^.

The major strength of this study is that, to the best of our knowledge, it is the first to analyze risk factors for liver biopsy-proven significant liver fibrosis in MAFLD patients with HBV infection. However, this study has some limitations, including its retrospective design. Although HOMA-IR and SCRP are mentioned in the diagnostic criteria for lean and normal-weight patients with MAFLD under the new definition, these were absent in our data, which may have caused us to miss some MAFLD cases. Second, given the cross-sectional nature of the study, we were unable to determine the causal relationship between metabolic dysfunction and significant liver fibrosis, highlighting the need for further longitudinal cohort studies to verify the effects of metabolic and viral factors on liver fibrosis in MAFLD patients with HBV infection. Furthermore, due to the gender distribution of MAFLD patients, the number of women included in this study was low; therefore, conclusions may pertain more to male patients, and further research is required to confirm these findings in females.

In conclusion, MAFLD patients with HBV infection have a higher risk of liver fibrosis than patients who have pure MAFLD. Metabolic factors, hypertension and type 2 diabetes, are closely related to liver fibrosis in MAFLD patients with HBV. These results highlight that in addition to traditional antiviral therapy, screening and early intervention of metabolic diseases are required for MAFLD patients with HBV infection. For patients with diabetes and hypertension, blood noninvasive biomarkers or transient elastography should be actively performed to further define the stage of liver fibrosis. If noninvasive screening presents a high risk of liver fibrosis, liver biopsy is recommended.

## Data Availability

The datasets used and/or analyzed during the current study are available from the corresponding author upon reasonable request.
